# Glutamine relieves feed restriction-induced ruminal epithelial function damage through histone lysine lactylation in yaks

**DOI:** 10.1186/s40104-025-01305-7

**Published:** 2025-12-18

**Authors:** Ziqi Yue, Liyuan Shi, Zhisheng Wang, Rui Hu, Quanhui Peng, Huawei Zou, Jianxin Xiao, Yahui Jiang, Fali Wu, Yiping Tang

**Affiliations:** 1 Animal Nutrition Institute, Sichuan Agricultural University, Chengdu, 611130 China; 2 Low Carbon Breeding Cattle and Safety Production University Key Laboratory of Sichuan Province, Sichuan Agricultural University, Chengdu, 611130 China

**Keywords:** Feed restriction, Glutamine, Glutamine deficiency, Histone lysine lactylation, Rumen epithelial cells, Yak

## Abstract

**Background:**

As a unique livestock adapted to the harsh environment, grazing yaks frequently suffer from malnutrition and even death because of the lower yield and quality of forage in the Qinghai-Tibet Plateau during the cold season. Certain stress conditions, such as environmental changes, disease, and malnutrition, can lead to a decrease in glutamine (Gln) synthesis, which fails to cover the physiological needs of the organism. Supplementation with exogenous Gln can promote nutrient digestion and improve rumen fermentation in ruminant animals under malnutrition. However, whether Gln could alleviate the barrier function injury induced by malnutrition and its mechanism is still unclear.

**Methods:**

In the in vivo experiments, 24 healthy yaks (31 months, 265.35 ± 25.81 kg) were randomly divided into 3 groups, namely control group (Con, free access to the basal diet), feed restriction group (FR, 50% level of ad libitum feed intake), and feed restriction + Gln group (FR + Gln, 50% level of ad libitum feed intake from d 1 to 30, 50% level of ad libitum feed intake + 1% Gln from d 31 to 60). In the in vitro experiments, the yak rumen epithelial cells (YRECs) were divided into 4 groups: Con group (complete medium), Gln group (complete medium + 10 mmol/L Gln), Gln deficiency group (Gln-D, Gln-free medium), and Gln deficiency + Gln group (Gln-D + Gln, Gln-free medium + 10 mmol/L Gln).

**Results:**

In the in vivo experiments, FR significantly decreased the ruminal concentrations of acetate, propionate, butyrate, iso-butyrate, and total volatile fatty acid (VFA) (*P* < 0.05). FR also reduced the mRNA expression of *NHE1*, *Na*^+^*/K*^+^*-ATPase*, and *Ca*^*2*+^*/Mg*^*2*+^*-ATPase*, and the concentrations of lactate, histone acetyltransferase (p300), histone deacetylase (HDAC), as well as the histone lysine lactylation level compared to Con group, while Gln supplementation alleviated them (*P* < 0.05). In the in vitro experiments, Gln alleviated the Gln-D-induced down-regulation of *NHE1*, *Na*^+^*/K*^+^*-ATPase*, and *Ca*^*2*+^*/Mg*^*2*+^*-ATPase* mRNA expressions and reduction of lactate, p300, HDAC concentrations, and histone lysine lactylation level (*P* < 0.05). Besides, p300 inhibitor abrogated Gln repair of barrier function damage in YRECs (*P* < 0.05).

**Conclusions:**

Overall, our results revealed the potential mechanism of Gln supplementation to repair malnutrition-induced damage of rumen epithelial barrier function in yaks, which might be related to histone lysine lactylation. However, because we do not have a control group receiving glutamine alone, we cannot determine the impact of Gln on the rumen epithelial function of normal yaks.

**Supplementary Information:**

The online version contains supplementary material available at 10.1186/s40104-025-01305-7.

## Introduction

As a unique livestock adapted to the harsh environment of the Qinghai-Tibet Plateau, the yak (*Bos grunniens*) can provide the basic resources for the local nomadic pastoralists [1]. Nevertheless, the particular natural environment in the Qinghai-Tibet Plateau resulted in a significantly lower yield and quality of forage during the cold season [2]. Therefore, grazing yaks frequently suffer from malnutrition, resulting in growth-retarded yaks and even death [3]. The rumen is the primary location for nutrient digestion and absorption in ruminants, and the rumen epithelium is essential for nutrient absorption, transport, and safeguarding the rumen wall [4, 5]. Previous studies have found that the apparent crude protein (CP) digestibility, volatile fatty acid (VFA) concentrations, and the protein and mRNA expression of tight junction in ruminal epithelial cells of growth-retarded yaks were significantly lower than that in growth-normal yaks, suggesting an impaired ruminal epithelial barrier function [6–8]. However, the effect of feed shortage on the rumen epithelial cell function of yaks still requires further research.

Glutamine (Gln), the most abundant free amino acid in mammals, is considered a “conditionally” necessary amino acid [9, 10]. Gln synthesis decreases and fails to cover the physiological needs of the organism under certain stress conditions, such as environmental changes, disease, and malnutrition [11]. In fact, Gln has been proven to enhance the rumen digestive enzyme activities and nutrient apparent digestibility, thereby promoting nutrient digestion and improve the rumen fermentation in ruminant animals under malnutrition [7, 12]. In addition, Gln plays a crucial role in maintaining the cell function of the rumen and intestinal epithelium [3, 13]. It has been found that starvation could reduce glutamine synthetase activity in the intestine of rats [14]. It indicates that starvation could decrease the Gln concentration. In the in vitro experiments, we indicated that Gln deficiency enhanced the expression of pro-inflammatory factors and decreased the expression of tight junctions in yak rumen epithelial cells [15]. Besides, the exogenous addition of Gln could maintain the epithelial cell function of the gastrointestinal tract under stress conditions. For example, Gln supplementation could alleviate heat stress-induced damage to intestinal epithelial barrier function in broilers [16]. Similarly, Gln supplementation increased the protein and mRNA expression of tight junction in rumen epithelial cells, thereby maintaining the integrity of rumen epithelial barrier in growth-retarded yaks [3]. Besides, a previous study revealed that Gln could repair the barrier function damage of yak rumen epithelial cells induced by histamine in vitro [17]. Thus, we want to explore the potential mechanism of Gln alleviating malnutrition-induced functional damage in the rumen epithelium.

Histone lysine lactylation, a histone modification first discovered in 2019, uses lactate as a modification donor to regulate gene expression [18]. Research has found that the inhibition of lactate dehydrogenase (LDH) activity reduced the lactate concentration and histone lysine lactylation level, thereby reducing the expression of downstream genes [18]. Meanwhile, histone lysine lactylation is modulated by histone acetyltransferase (p300) and histone deacetylase (HDAC) 1–3. The level of histone lysine lactylation increased by lactate was diminished in p300-knockdown macrophages [19]. Compared to only knockdown of HDAC1 or HDAC3, knockdown of HDAC1–3 resulted in a stronger effect on histone lysine lactylation [20]. A previous study found that lactate could increase the level of histone lysine lactylation in mouse intestinal epithelial cells, thereby inducing the inflammatory bowel disease (IBD) [21]. IBD has been proven to impair epithelial barrier function. A study on ruminants showed that the suitable lactate concentration increased the histone lysine lactylation level and participated in regulating redox homeostasis, cell adhesion, cell proliferation, and apoptosis in the endometrium of sheep [22]. A previous study revealed that LDHA activity and lactate concentration were significantly decreased in the rumen epithelial of growth-retarded yaks compared to growth-normal yaks [23]. Moreover, existing research has proven rumen epithelial cells can utilize Gln to generate lactate and are regulated by nutritional intake [24]. Consequently, in yak rumen epithelial cells, whether lactate produced from Gln can regulate histone lysine lactylation remains to be researched.

In summary, we hypothesized that histone lysine lactylation is involved in the mitigation of malnutrition-induced cell function damage to rumen epithelial cells in yaks by Gln. To test our hypothesis, we studied the molecular mechanism using in vitro and in vivo experiments. This study is expected to offer theoretical guidance for the further promotion and application of Gln in the yak industry.

## Materials and methods

### Ethics statement

Animal experimental was approved by the Animal Care and Use Committee of Sichuan Agricultural University (YZQ-2021114009). According to State Council Decree No. 676, Regulations on the Administration of Laboratory Animals (2017 Revision) were followed for the animal experiment.

### Animals, experimental design and diets

This study was implemented at the Ya’an experimental site of the Animal Nutrition Institute, Sichuan Agricultural University. In this study, 24 healthy female yaks, 31-month-old, with similar body weight (265.35 ± 25.81 kg) were selected. The study spanned 75 d, with a 15-day initial feeding phase for adaptation (−15 to 0 d) followed by a 60-day experimental feeding phase (1 to 30 d and 31 to 60 d two stages). All yaks were randomly divided into 3 groups with 8 replicates per group and one yak per replicate: Control (Con) group, feed restriction (FR) group, and feed restriction + glutamine (FR + Gln) group. From d −15 to 0, all yaks in three groups were given basal diet ad libitum. From d 1 to 30, yaks in Con group had free access to the basal diet, yaks in FR and FR + Gln groups were fed a 50% basal diet of the dry matter intake (DMI) of the adaptation period. From d 31 to 60, yaks in Con group had free access to the basal diet, yaks in FR group were fed a 50% basal diet of the DMI of the adaptation period, yaks in FR + Gln group were fed a 50% basal diet of the DMI of the adaptation period and 1% Gln (99% purity, chx-51, Fufeng, Shandong, China). The yaks received feedings twice a day at 8:00 and 16:00, with unlimited access to water. The basal diet was formulated according to the Chinese Beef Cattle Raising Standard (Feeding standard of beef cattle NY/T 815–2004) and mixed as total mixed ration (TMR). The feed compositions and nutrient levels of the basal diets are shown in Table S1 [25]. 

### Sample collection and measurement

#### Diet, leftover feed, and faeces samples

A 5-day digestion and metabolism test (61 to 65 d) was conducted at the end of the formal period. During this period, the diet, leftover feed, and faeces samples were collected from 24 yaks in three groups. Among them, the diet samples (approximately 100 g) and the leftover feed samples (approximately 100 g) from the previous day were collected daily before morning feeding and stored at −20 °C. Afterward, the 5-day diet samples were thoroughly mixed, and the 5-day leftover feed samples from each yak were thoroughly mixed to produce a composite sample and stored at −20 °C. At the same time, faeces samples were collected four times a day by rectal sampling, with collection times at 09:00, 15:00, 21:00, and 03:00 (approximately 300 g each time). After each sampling, each 100 g fresh faeces sample was mixed with 10 mL of 10% sulfuric acid and frozen at −20 °C. Subsequently, 5-day faeces samples from each yak were thoroughly mixed to produce a composite sample (approximately 6,000 g), and new faeces samples (approximately 600 g) were collected. After experiment, the diet, leftover feed, and faeces samples were dried at 65 °C for 48 h to a constant weight and pulverized for subsequent analysis.

#### Rumen fluid

On d 60, the rumen fluid was collected from each yak using the oro-esophageal probe rumen sampler before morning feeding. To prevent contamination of ruminal fluid by saliva or mucus, the initial 150 mL of ruminal fluid was discarded before collecting another 100 mL. Then, the rumen fluid samples were filtered with 4 layers of gauze, dispensed into the 15-mL centrifuge tube, and stored at −20 °C for rumen fermentation analysis.

#### Rumen epithelial tissue

After all experiment, 6 yaks were randomly selected from each group for humanely slaughtered following the National Standard Operating Procedures (GB/T 19477–2018, cattle slaughtering, China). Then, the rumen epithelial tissue was collected after the digesta was removed. For molecular determination, the rumen epithelial tissue was sliced into pieces and washed with phosphate buffered saline (PBS), divided into the 2-mL cryopreservation tube, immediately stored in liquid nitrogen, and then transported to the research laboratory and stored at −80 °C.

### Cell culture and treatment

The yak rumen epithelial cells (YRECs) cell line involved in our research was established in our laboratory and obtained an invention patent (Patent Number: LU502505) [26]. Complete medium was composed of 89% RPMI-1640 (C22400500BT, Gibco, Shanghai, China) medium, 10% fetal bovine serum (FBS, 10099-141, Gibco), and 1% penicillin–streptomycin-amphotericin (G4015, Servicebio, Wuhan, China). The YRECs culture condition was at 37 °C with 5% CO_2_. YRECs used in our research were between passages 20 and 30. According to previous study [15], the complete medium was exchanged for serum replacement (SR) medium in our research. The SR medium was composed of 89% RPMI-1640 medium, 10% SR (Gln-free, C10828028BT, Gibco), and 1% penicillin–streptomycin-amphotericin. In addition, our research also used the Gln-free medium. The Gln-free medium was composed of 89% Gln-free RPMI-1640 medium (C21870076BT, Gibco), 10% SR, and 1% penicillin–streptomycin-amphotericin.

According to previous studies [15, 27], our research involved 4 treatment groups: Control (Con) group, Gln group, Gln deficiency (Gln-D) group, and Gln deficiency + Gln (Gln-D + Gln) group. Among those, YRECs in Con group were cultured with the SR medium for 12 h and cultured for another 24 h in a fresh SR medium. YRECs in Gln group were cultured with the SR medium for 12 h and cultured for another 24 h in a fresh SR medium supplemented with 10 mmol/L Gln (99% purity, G8230, Solarbio, Beijing, China). YRECs in Gln-D group were cultured with the Gln-free medium for 12 h and cultured for another 24 h in a fresh Gln-free medium. YRECs in Gln-D + Gln group were cultured with the Gln-free medium for 12 h and cultured for another 24 h in a fresh Gln-free medium supplemented with 10 mmol/L Gln.

In order to observe the critical role of histone lysine lactylation in Gln alleviation of Gln-D-induced injury in YRECs. We used the LDHA inhibitor (GEN-140, HY-100742, MCE, Shanghai, China), p300 inhibitor (C646, HY-13823, MCE), and HDAC inhibitor (SAHA, HY-10221, MCE). On the base of the above preliminary experiments, we added three groups: Gln-D + Gln + GEN-140 group, Gln-D + Gln + C646 group, and Gln-D + Gln + SAHA group. YRECs in Gln-D + Gln + GEN-140 group were cultured with the Gln-free medium for 12 h and cultured for another 24 h in a fresh Gln-free medium supplemented with 10 mmol/L Gln and 5 µmol/L Gen-140. YRECs in Gln-D + Gln + C646 group were cultured with the Gln-free medium for 12 h and cultured for another 24 h in a fresh Gln-free medium supplemented with 10 mmol/L Gln and 10 µmol/L C646. YRECs in Gln-D + Gln + SAHA group were cultured with the Gln-free medium for 12 h and cultured for another 24 h in a fresh Gln-free medium supplemented with 10 mmol/L Gln and 5 µmol/L SAHA.

### Measurement indicators

#### Diet, leftover feed, and faeces analyses

The dry matter (DM, 105 °C) content and crude fiber (CF, 978.10) content in diet, leftover feed, and faeces samples were determined according to AOAC [28]. The neutral detergent fiber (NDF) content and acid detergent fiber (ADF) content were determined according to Van Soest et al. [29]. Both NDF and ADF values were expressed inclusive of residual ash.

#### Rumen fermentation

The total VFA, acetate (A), propionate (P), butyrate, iso-valerate, and iso-butyrate were analyzed using the gas chromatograph (Varian CP-3800 GC, USA) according to previous study [30]. Briefly, the rumen fluid was centrifuged to obtain the supernatant, which was then mixed with metaphosphoric acid and crotonic acid. Subsequently, the mixture was incubated at 4 °C for 30 min and centrifuged to collect the new supernatant. After dilution with chromatographic-grade methanol, the concentrations of VFA were determined using a gas chromatograph. Finally, the acetate/propionate ratio (A/P) was calculated.

#### Real-time quantitative PCR (RT-qPCR)

To determine the mRNA expression levels, the RT-qPCR was performed. The total RNA of rumen epithelial tissue and YRECs was extracted using the SteadyPure Quick RNA Extraction kit (AG21101, Accurate Biology, Changsha, China). And a Reverse Transcription Kit (A502-01, Exongen, Chengdu, China) was used to reverse-transcribe the total RNA to cDNA. Afterward, q-PCR was performed using a real-time q-PCR system (QuantStudio 5, USA) with the SYBR Green q-PCR Master Mix kit (G3328, Servicebio). Glyceraldehyde-3-phosphate dehydrogenase (GAPDH) was chosen as our internal control. Then, analysis of real-time PCR data was performed using the 2^−^^ΔΔCT^ method. The primer sequences for quantitation of target genes were shown in Table S2.

#### Enzyme activity or metabolite concentration

The enzyme activities of Na^+^/K^+^-ATPase (KTB1800, Abbkine, Wuhan, China), Ca^2^^+^/Mg^2^^+^-ATPase (KTB1810, Abbkine), LDHA (MM-51344O1, Jiangsu Meimian industrial Co., Ltd., Jiangsu, China), p300 (MM-1054V1, Jiangsu Meimian industrial), and HDAC1–3 (HDAC1, MM-1052V1, HDAC2, MM-1058V1, HDAC3, MM-1047V1, Jiangsu Meimian industrial) and the concentrations of lactate (LA; A019-2-2, Nanjing Jiancheng Bioengineering Institute, Jiangsu, China), Gln (MM-5115701, Jiangsu Meimian industrial), glutamate (Glu; KTB1440, Abbkine), α-ketoglutaric acid (α-KG; BC5425, Solarbio), reduced glutathione (GSH; KTB1600, Abbkine), glutaminase enzyme (GlnS; MM-1039V2, Jiangsu Meimian industrial), glutamate dehydrogenase (GDH; MM-1043V2, Jiangsu Meimian industrial), and γ‐glutamyl-cysteine ligase (GCL; MM-1021V2, Jiangsu Meimian industrial) were detected according to the manufacturers’ instructions.

#### Western blot

The RIPA lysis buffer (P0013B, Beyotime, Shanghai, China) was used to extract the total protein from rumen epithelial tissue and YRECs. The total protein concentration was detected using a bicinchoninic acid (BCA) protein assay kit (KTD3001, Abbkine). Then, 20 μg proteins were isolated for western blot analysis. Briefly, the proteins were separated using the sodium dodecyl sulfate-polyacrylamide gel electrophoresis (SDS-PAGE) and electrophoretically transferred onto polyvinylidene difluoride (PVDF) membranes. Primary antibodies were incubated overnight, and secondary antibodies were incubated for 2 h. The protein blot was finally obtained through the use of the enhanced chemiluminescence developer (PD203, Oriscience, Chengdu, China) and chemiluminescence system (Touch Imager Pro, AGP2307Y05, Shanghai, China), and analyzed via ImageJ v1.80 software (NIH, Bethesda, MD, USA). The antibody information is given in Table S3.

#### Transepithelial electrical resistance

The YRECs were seeded at a density of 1 × 10^5^ on 12-well polystyrene Transwell filters with 0.4 μm pore size and 1.12-cm^2^ growth area (Corning Costar, NY, USA). The transepithelial electrical resistance (TEER) was measured using the Millicell^®^-ERS (Millipore, Billerica, MA, USA) and assessed the cell monolayer integrity. The TEER was measured daily after YRECs seeding until the TEER stabilized, after which experimental treatments. Then, the TEER in four groups was measured at 12 h after the Gln-D treatment (This time point was defined as 0 h). After that, the TEER was measured at 3 h, 6 h, 12 h, and 24 h after the Gln supplementation treatment. No cells were inoculated in the blank group. The calculation formula was TEER (Ω·cm^2^) = (test group resistance value – blank resistance value) × transwell culture chamber membrane area.

### Statistical analysis

In vivo study, the results were analyzed by analysis of variance (ANOVA) followed by Tukey's multiple comparisons (SPSS 27.0, IBM, Chicago, IL, USA). The mathematical model is expressed as: *Y*_*ij*_ = *μ* + *T*_*i*_ + *J*_*i*_ + *e*_*ij*_, where *Y*_*ij*_ is the observation of dependent variables; *μ* is the overall mean; *T*_*i*_ is the fixed effect of treatment (dietary); *J*_*i*_ is the random effect of animal ID; e_*ij*_ is the random error. In vitro study, the results were analyzed by one-way ANOVA followed by Tukey's multiple comparisons (SPSS 27.0, IBM, Chicago, IL, USA). All graphs were created using the GraphPad Prism 8.0 software (GraphPad, La Jolla, CA, USA). The data were expressed as arithmetic mean ± standard error of the mean (mean ± SEM). A significant difference was indicated by *P* < 0.05.

## Results

### Nutrient apparent digestibility

The nutrient apparent digestibility of the yaks is summarized in Table 1. Compared with the Con group, FR did not significantly affect the digestibility of DM, NDF, ADF, and CF (*P* > 0.05). However, dietary Gln supplementation significantly increased the apparent digestibility of DM (*P* = 0.049), ADF (*P* = 0.022), NDF (*P* = 0.001), and CF (*P* = 0.001) compared to the FR group.

**Table 1:** Effects of dietary Gln supplementation on nutrient apparent digestibility of feed-restricted yaks (*n* = 8)

Items	Con	FR	FR + Gln	SEM	*P*-value
DM, %	60.42^b^	59.52^b^	62.92^a^	0.316	0.049
NDF, %	57.19^b^	57.04^b^	60.11^a^	0.033	0.022
ADF, %	38.14^b^	37.55^b^	44.67^a^	0.046	0.001
CF, %	36.80^b^	38.20^b^	45.17^a^	0.519	0.001

*Con *Control group, *FR *Feed restriction group, *FR + Gln *Feed restriction + Gln group, *SEM *Standard error of the mean, *DM *Dry matter, *NDF *Neutral detergent fiber, *ADF *Acid detergent fiber, *CF *Crude fiber. Results were expressed as mean with SEM

^a,b^Within a row, means with different superscripts indicated significantly different (*P* < 0.05)

### Rumen fermentation parameters

As shown in Table 2, the ruminal concentrations of total VFA (*P* < 0.001), acetate (*P* = 0.013), propionate (*P* = 0.002), butyrate (*P* = 0.003), and iso-butyrate (*P* = 0.03) of yaks in the FR group were less than the yaks in the Con group. Compared with the FR group, FR + Gln significantly increased the concentrations of total VFA (*P* < 0.001), acetate (*P* = 0.013), and butyrate (*P* = 0.003), but had no significant effect on the concentrations of propionate and iso-butyrate (*P* > 0.05). Furthermore, FR and FR + Gln had no significant effect on the iso-valerate concentration (*P* = 0.128).

**Table 2:** Effects of dietary Gln supplementation on rumen fermentation parameters of feed-restricted yaks (*n* = 8)

Items	Con	FR	FR + Gln	SEM	*P*-value
Total VFA, mmol/L	79.73^a^	58.62^b^	80.15^a^	3.228	< 0.001
Acetate (A), mmol/L	56.64^a^	44.55^b^	61.60^a^	5.405	0.013
Propionate (P), mmol/L	16.43^a^	10.19^b^	10.31^b^	1.744	0.002
Butyrate, mmol/L	4.63^b^	2.32^c^	6.62^a^	1.004	0.003
Iso-valerate, mmol/L	0.98	0.80	0.81	0.098	0.128
Iso-butyrate, mmol/L	1.05^a^	0.76^b^	0.81^b^	0.110	0.030
A:P	3.83^b^	4.22^ab^	4.75^a^	0.237	0.003

*Con *Control, *FR *Feed restriction, *FR + Gln *Feed restriction + Gln, *SEM *Standard error of the mean, *VFA *Volatile fatty acid, *A:P *Acetate to propionate ratio. Results were expressed as mean with SEM

^a–c^ Within a row, means with different superscripts indicated significantly different (*P* < 0.05)

### Ion transporters, ATPase, and histone lysine lactylation in rumen epithelium

**Fig. 1:**
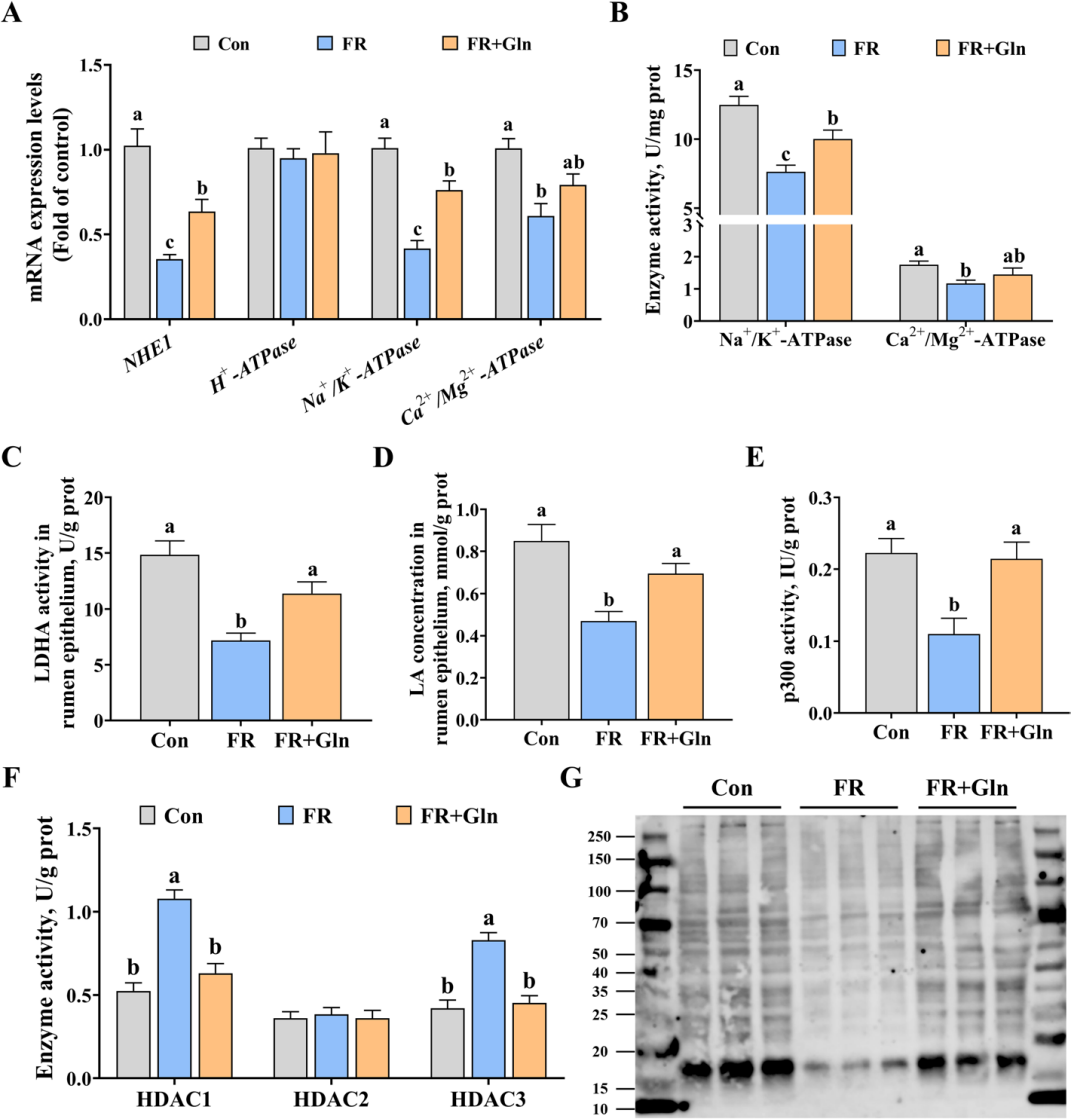
Effects of feed restriction and dietary Gln supplementation on ion transporter, ATPase, and histone lysine lactylation in rumen epithelium of yaks. **A** The mRNA levels of *NHE1*, *H*^+^*-ATPase*, *Na*^+^*/K*^+^*-ATPase*, and C*a*^*2*+^*/Mg*^*2*+^*-ATPase*. **B** The enzyme activities of Na^+^/K^+^-ATPase and Ca^2+^/Mg^2+^-ATPase. **C** The enzyme activity of LDHA. **D** LA concentration. **E** The enzyme activity of p300. **F** The enzyme activities of HDAC1, HDAC2, and HDAC3. **G** Histone lysine lactylation levels (Pan lactylation). Results were expressed as means ± SEM, *n* = 6 independent experiments. ^a–c^ Different letters indicate significant differences (*P* < 0.05). Con = control group; FR = feed restriction group; FR + Gln = feed restriction + Gln group; NHE1 = sodium proton exchanger 1; LDHA = lactate dehydrogenase A; LA = lactate; p300 = histone acetyltransferase; HDAC = histone deacetylase; prot = protein

The effect of FR and FR + Gln on ion transporters, ATPase, and histone lysine lactylation in rumen epithelium of yaks were further evaluated. Firstly, as shown in Fig. 1A and B, the mRNA expression of *NHE1* (*P* < 0.001), *Na*^+^*/K*^+^*-ATPase* (*P* < 0.001), and *Ca*^*2*+^*/Mg*^*2*+^*-ATPase* (*P* = 0.002) and the activities of Na^+^/K^+^-ATPase (*P* < 0.001) and Ca^2+^/Mg^2+^-ATPase (*P* = 0.029) were significantly lower in the FR group than in the Con group. However, compared with the FR group, FR + Gln significantly increased the activity of Na^+^/K^+^-ATPase (*P* = 0.031) and the mRNA expression of *NHE1* (*P* = 0.036) and *Na*^+^*/K*^+^*-ATPase* (*P* = 0.001), but had no significant effect on the mRNA expression of *Ca*^*2*+^*/Mg*^*2*+^*-ATPase* (*P* = 0.151) and the activity of Ca^2+^/Mg^2+^-ATPase (*P* = 0.37). Furthermore, FR and FR + Gln had no significant effect on the expression of *H*^+^*-ATPase* (*P* = 0.888).

Secondly, as shown in Fig. 1C–F, compared with the Con group, FR significantly reduced the LDHA activity (*P* < 0.001), LA concentration (*P* = 0.001), p300 activity (*P* = 0.006) and increased the HDAC1 activity (*P* < 0.001) and HDAC3 activity (*P* < 0.001), while FR + Gln alleviated them (*P* < 0.05). In addition, western blot analysis showed that FR significantly reduced the histone lysine lactylation compared to the Con group, while FR + Gln alleviated it (Fig. 1G).

### Barrier function, ion transporter, and ATPase in YRECs

**Fig. 2:**
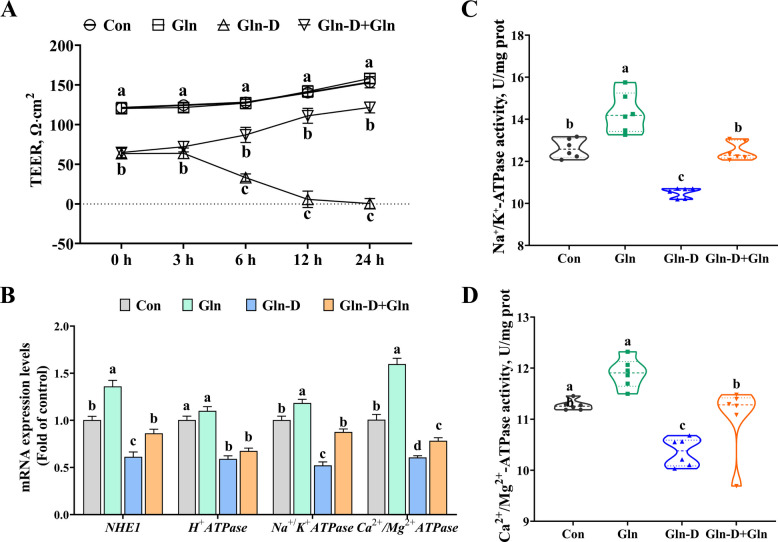
Effects of Gln supplementation on barrier function, ion transporter, and ATPase of Gln-deficiency YRECs. **A** The transepithelial electrical resistance. **B** The mRNA levels of *NHE1*, *H*^+^*-ATPase*, *Na*^+^*/K*^+^*-ATPase*, and C*a*^*2*+^*/Mg*^*2*+^*-ATPase*. **C** and **D** The enzyme activities of Na^+^/K^+^-ATPase and Ca^2+^/Mg^2+^-ATPase. Results were expressed as means ± SEM, *n* = 6 independent experiments. ^a–c^ Different letters indicate significant differences (*P* < 0.05). Con = control group; Gln = glutamine group; Gln-D = Gln deficiency group; Gln-D + Gln = Gln deficiency + Gln group; TEER = transepithelial electrical resistance; NHE1 = sodium proton exchanger 1; YRECs = yak rumen epithelial cells; prot = protein

In this study, we discovered that Gln-D significantly decreased the value of TEER in YRECs (*P* < 0.05) (Fig. 2A). However, compared with the Gln-D group, the TEER value was significantly increased after Gln supplementation at 6 h, 12 h, and 24 h (*P* < 0.05) (Fig. 2A). In addition, as presented in Fig. 2B–D, Gln-D significantly reduced the mRNA expression of *NHE1* (*P* < 0.001), *H*^+^*-ATPase* (*P* < 0.001), *Na*^+^*/K*^+^*-ATPase* (*P* < 0.001), and *Ca*^*2*+^*/Mg*^*2*+^*-ATPase* (*P* < 0.001) and the activities of Na^+^/K^+^-ATPase (*P* < 0.001) and Ca^2+^/Mg^2+^-ATPase (*P* = 0.003) compared to the Con group. Nevertheless, compared with the Gln-D group, Gln-D + Gln significantly increased the mRNA expression of *NHE1* (*P* = 0.011), *Na*^+^*/K*^+^*-ATPase* (*P* < 0.001), and *Ca*^*2*+^*/Mg*^*2*+^*-ATPase* (*P* = 0.041) and the activities of Na^+^/K^+^-ATPase (*P* < 0.001) and Ca^2+^/Mg^2+^-ATPase (*P* = 0.033).

### Glutamine metabolism in YRECs

**Fig. 3:**
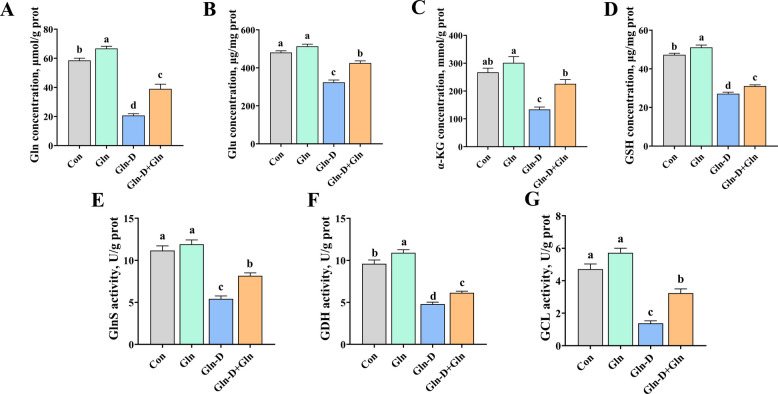
Effects of Gln supplementation on glutamine metabolism of Gln-deficiency YRECs. **A** Gln concentration. **B** Glu concentration. **C** α-KG concentration. **D** GSH concentration. **E** GlnS activity. **F** GDH activity. **G** GCL activity. Results were expressed as means ± SEM, *n* = 6 independent experiments. ^a–c^ Different letters indicate significant differences (*P* < 0.05). Con = control group; Gln = glutamine group; Gln-D = Gln deficiency group; Gln-D + Gln = Gln deficiency + Gln group; Gln = glutamine; Glu = glutamate; α-KG = α-ketoglutaric acid; GSH = reduced glutathione; GlnS = glutaminase enzyme; GDH = glutamate dehydrogenase; GCL = γ‐glutamyl-cysteine ligase; prot = protein

As shown in Fig. 3, the concentration of Gln, Glu, α-KG, and GSH and the activities of GlnS, GDH, and GCL were significantly lower in Gln-D group than Con group (*P* < 0.001). In contrast, Gln-D + Gln treatment increased the concentration of Gln (*P* < 0.001), Glu (*P* < 0.001), α-KG (*P* = 0.026), and GSH (*P* = 0.003) and the activities of GlnS (*P* = 0.002), GDH (*P* = 0.042), and GCL (*P* < 0.001) compared to the Gln-D group.

### Histone lysine lactylation in YRECs

**Fig. 4:**
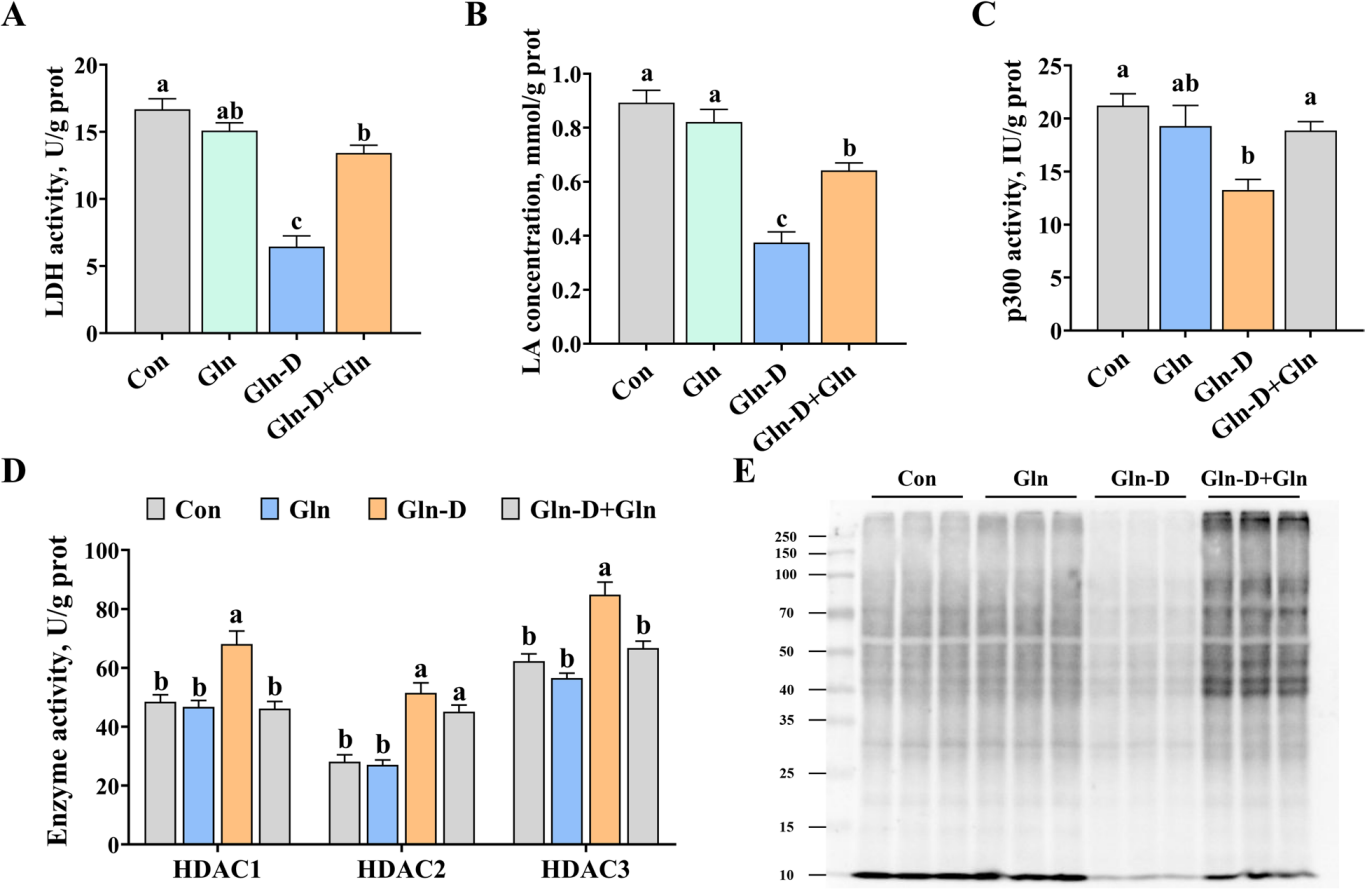
Effects of Gln supplementation on histone lysine lactylation of Gln-deficiency YRECs. **A** LDHA activity. **B** LA concentration. **C** p300 activity. **D** The enzyme activities of HDAC1, HDAC2, and HDAC3. **E** Histone lysine lactylation levels (Pan lactylation). Results were expressed as means ± SEM, *n* = 6 independent experiments. ^a–c^ Different letters indicate significant differences (*P* < 0.05). Con = control group; Gln = glutamine group; Gln-D = Gln deficiency group; Gln-D + Gln = Gln deficiency + Gln group; LDHA = lactate dehydrogenase A; LA = lactate; p300 = histone acetyltransferase; HDAC = histone deacetylase; prot = protein

In vitro, we also investigate the impact of Gln-D and Gln supplementation on histone lysine lactylation in YRECs. As shown in Fig. 4A–D, Gln-D significantly reduced the LDHA activity (*P* < 0.001), LA concentration (*P* < 0.001), p300 activity (*P* = 0.002), and increased the HDAC1 (*P* < 0.001) and HDAC3 (*P* < 0.001) activities compared to Con group. However, compared with the Gln-D group, Gln-D + Gln significantly increased the LDHA activity (*P* < 0.001), LA concentration (*P* < 0.001), p300 activity (*P* = 0.03), and decreased the HDAC1 (*P* < 0.001) and HDAC3 (*P* = 0.001) activities. In addition, western blot analysis showed that Gln-D significantly reduced the histone lysine lactylation in YRECs compared to the Con group, while Gln-D + Gln alleviated it (Fig. 4E).

### Effects of LDHA inhibitor, p300 inhibitor, and HDAC inhibitor on histone lysine lactylation of YRECs

**Fig. 5:**
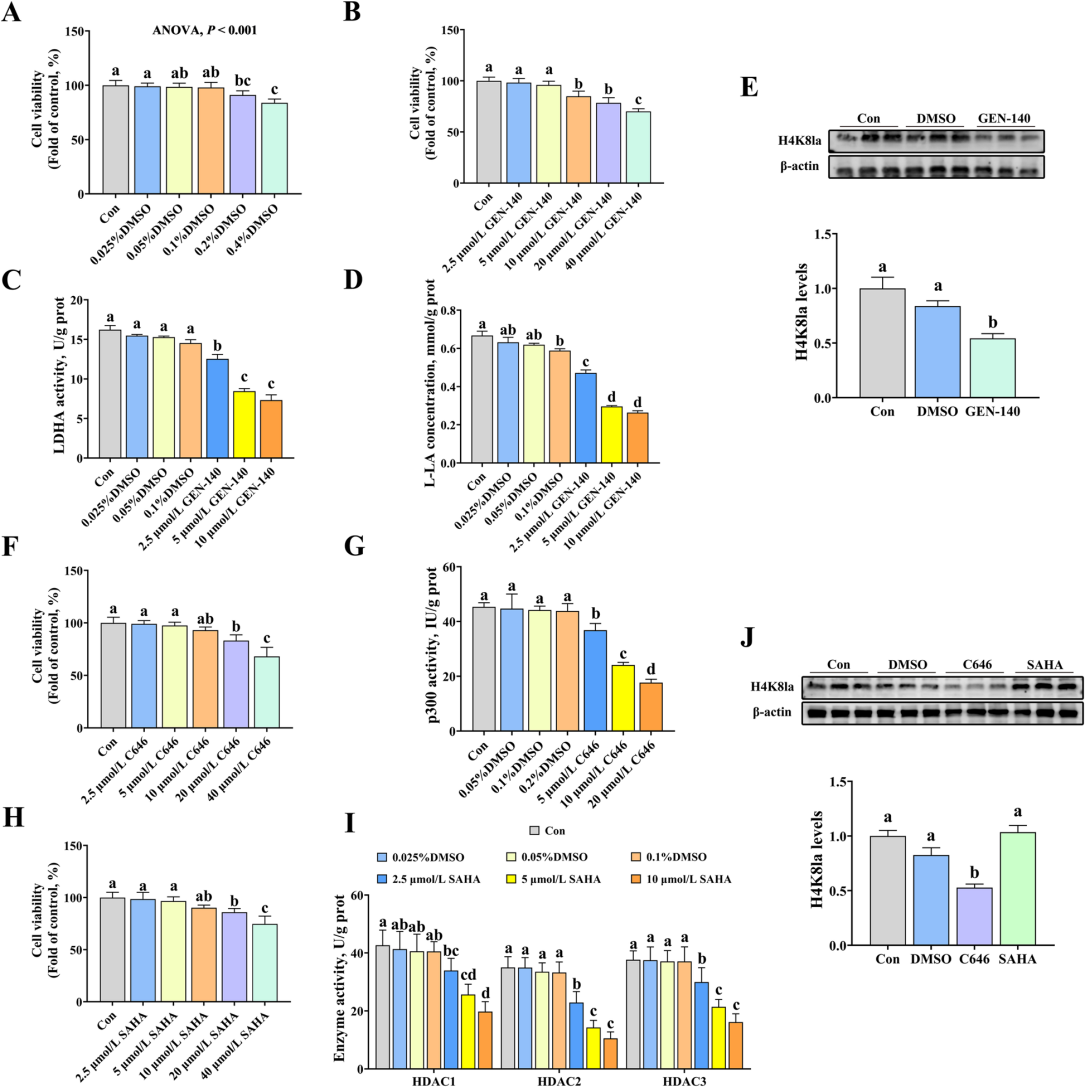
Effects of LDHA inhibitor, p300 inhibitor, and HDAC inhibitor on yak rumen epithelial cells. **A** Effect of different concentrations of DMSO (0, 0.025%, 0.05%, 0.1%, 0.2%, and 0.4%) on cell viability. **B** Effect of different concentrations of GEN-140 (LDHA inhibitor, 0, 2.5, 5, 10, 20, and 40 μmol/L) on cell viability. **C** Effect of different concentrations of DMSO (0.025%, 0.05%, 0.1%) and GEN-140 (2.5, 5, and 10 μmol/L) on LDHA activity. **D** Effect of different concentrations of DMSO (0.025%, 0.05%, 0.1%) and GEN-140 (2.5, 5, and 10 μmol/L) on LA concentration. **E** Effect of DMSO (0.05%) and GEN-140 (5 μmol/L) on H4K8la. **F** Effect of different concentrations of C646 (p300 inhibitor, 2.5, 5, 10, 20, and 40 μmol/L) on cell viability. **G** Effect of different concentrations of DMSO (0.05%, 0.1%, 0.2%) and C646 (5, 10, and 20 μmol/L) on p300 activity. **H** Effect of different concentrations of SAHA (2.5, 5, 10, 20, and 40 μmol/L) on cell viability. **I** Effect of different concentrations of DMSO (0.025%, 0.05%, 0.1%) and SAHA (2.5, 5, and 10 μmol/L) on HDAC (1, 2, and 3) activity. **J** Effect of DMSO (0.1%), C646 (10 μmol/L) and SAHA (5 μmol/L) on H4K8la. Results were expressed as means ± SEM, *n* = 6 independent experiments. ^a^^–^^c^ Different letters indicate significant differences (*P* < 0.05). Con = control group; Gln = glutamine group; Gln-D = Gln deficiency group; Gln-D + Gln = Gln deficiency + Gln group; NHE1 = sodium proton exchanger 1; LDHA = lactate dehydrogenase A; LA = lactate; p300 = histone acetyltransferase; HDAC = histone deacetylase; H4K8la = Histone H4 Lysine 8 Lactylation; DMSO = dimethyl sulfoxide

LDHA inhibitor, p300 inhibitor, and HDAC inhibitor were used to further examine the mechanism of Gln regulating ruminal epithelial cell function through the histone lysine lactylation. Firstly, the liquid chromatography-tandem mass spectrometry (LC-MS/MS) and protein identification analysis showed that histone H4 lysine 8 lactylation (H4K8la) was a primary modification site for Gln-D and Gln supplementation treatment (Tables S4–S7). In addition, we determined the appropriate concentration of LDHA inhibitor, p300 inhibitor, and HDAC inhibitor according to CCK-8 assay. The 5 μmol/L GEN-140, 10 μmol/LC646, and 5 μmol/L SAHA were the appropriate concentrations (Fig. 5).

**Fig. 6:**
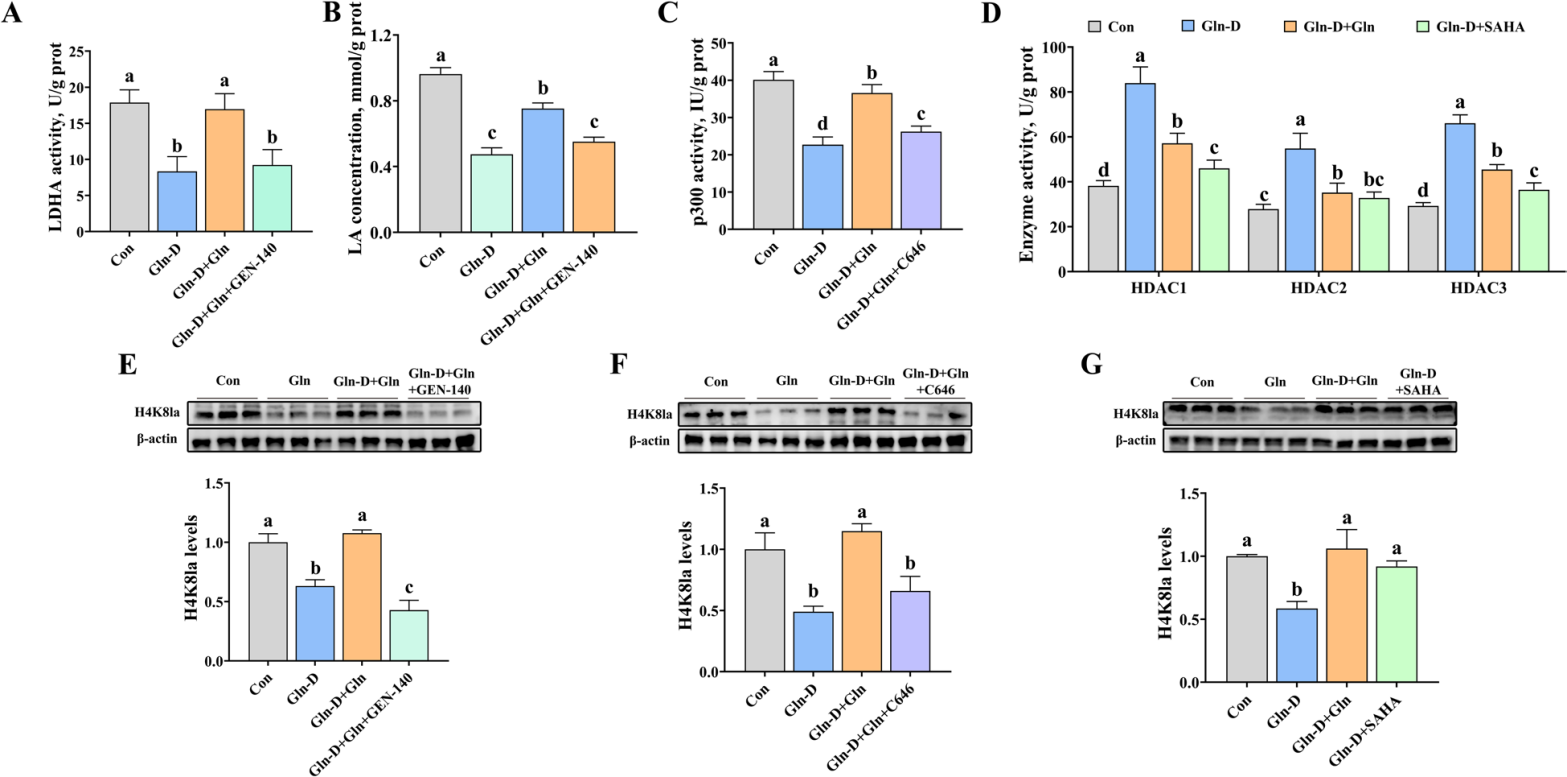
Effects of LDHA inhibitor, p300 inhibitor, and HDAC inhibitor on histone lysine lactylation of YRECs. **A** LDHA activity (LDHA inhibitor). **B** LA concentration (LDHA inhibitor). **C** p300 activity (p300 inhibitor). **D** The enzyme activities of HDAC1, HDAC2, and HDAC3 (HDAC inhibitor). **E** Histone lysine lactylation levels (Pan lactylation) (LDHA inhibitor). **F** Histone lysine lactylation levels (Pan lactylation) (p300 inhibitor). **G** Histone lysine lactylation levels (Pan lactylation) (HDAC inhibitor). Results were expressed as means ± SEM, *n* = 6 independent experiments. ^a–c^ Different letters indicate significant differences (*P* < 0.05). Con = control group; Gln-D = Gln deficiency group; Gln-D + Gln = Gln deficiency + Gln group; Gln-D + Gln + GEN-140 = Gln deficiency + Gln + GEN-140 (LDHA inhibitor) group; Gln-D + Gln + C646 = Gln deficiency + Gln + C646 (p300 inhibitor) group; Gln-D + Gln + SAHA = Gln deficiency + Gln + SAHA (HDAC inhibitor) group; LDHA = lactate dehydrogenase A; LA = lactate; p300 = histone acetyltransferase; HDAC = histone deacetylase; GEN-140 = LDHA inhibitor; C646 = p300 inhibitor; SAHA = HDAC inhibitor; H4K8la = Histone H4 Lysine 8 Lactylation; prot = protein

Secondly, we studied the effect of the LDHA inhibitor, p300 inhibitor, and HDAC inhibitor on histone lysine lactylation in YRECs. As shown in Fig. 6A, B, and E, compared with the Gln-D + Gln group, Gln-D + Gln + GEN-140 (LDHA inhibitor) significantly decreased the LDHA activity (*P* < 0.001), LA concentration (*P* = 0.003), and histone H4K8la modification (*P* < 0.001). Similarly, Gln-D + Gln + C646 (p300 inhibitor) significantly decreased the p300 activity (*P* < 0.001) and H4K8la (*P* = 0.001) compared to Gln-D + Gln group (Fig. 5C and F). In addition, compared with the Gln-D group, Gln-D + SAHA (HDAC inhibitor) significantly decreased the activities of HDAC1 (*P* = 0.003), HDAC2 (*P* < 0.001), and HDAC3 (*P* < 0.001) and increased H4K8la (*P* = 0.005) (Fig. 6D and G).

### Effects of LDHA inhibitor, p300 inhibitor, and HDAC inhibitor on ion transporter, ATPase, and tight junction of YRECs

**Fig. 7:**
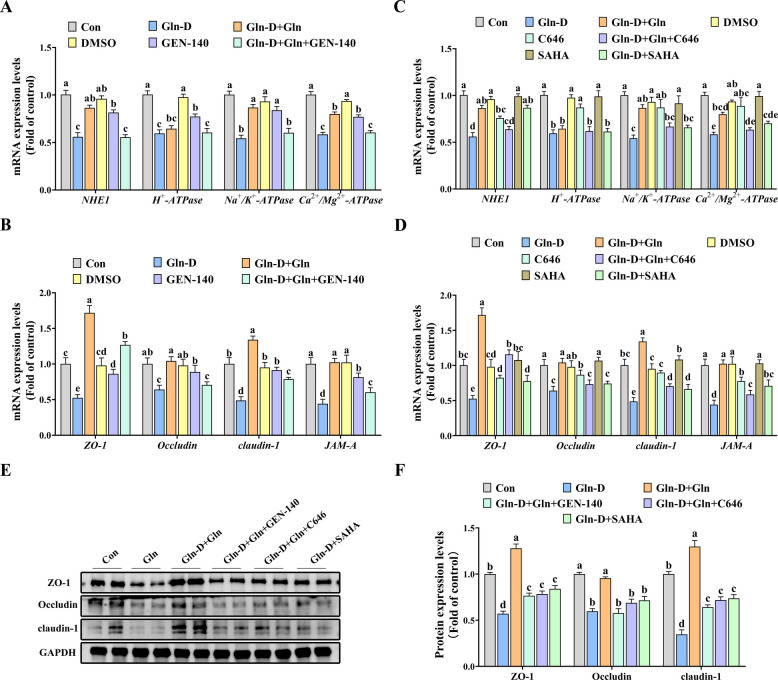
Effects of LDHA inhibitor, p300 inhibitor, and HDAC inhibitor on barrier function of YRECs. **A** The mRNA levels of *NHE1*, *H*^+^*-ATPase*, *Na*^+^*/K*^+^*-ATPase*, and C*a*^*2*+^*/Mg*^*2*+^*-ATPase* (LDHA inhibitor). **B** The mRNA levels of *NHE1*, *H*^+^*-ATPase*, *Na*^+^*/K*^+^*-ATPase*, and C*a*^*2*+^*/Mg*^*2*+^*-ATPase* (p300 inhibitor and HDAC inhibitor). **C** The mRNA levels of *ZO-1*, *Occludin*, *claudin-1*, and *JAM-A* (LDHA inhibitor). **D** The mRNA levels of *ZO-1*, *Occludin*, *claudin-1*, and *JAM-A* (p300 inhibitor and HDAC inhibitor). **E** and **F** The protein levels of ZO-1, Occludin, and claudin-1. Results were expressed as means ± SEM, *n* = 6 independent experiments. ^a–c^ Different letters indicate significant differences (*P* < 0.05). Con = control group; Gln-D = Gln deficiency group; Gln-D + Gln = Gln deficiency + Gln group; DMSO = dimethyl sulfoxide group; GEN-140 = LDHA inhibitor group; Gln-D + Gln + GEN-140 = Gln deficiency + Gln + GEN-140 (LDHA inhibitor) group; C646 = p300 inhibitor group; SAHA = HDAC inhibitor group; Gln-D + Gln + C646 = Gln deficiency + Gln + C646 (p300 inhibitor) group; Gln-D + Gln + SAHA = Gln deficiency + Gln + SAHA (HDAC inhibitor) group; DMSO = dimethyl sulfoxide; p300 = histone acetyltransferase; HDAC = histone deacetylase; GEN-140 = LDHA inhibitor; LDHA = lactate dehydrogenase A; C646 = p300 inhibitor; SAHA = HDAC inhibitor; NHE1 = sodium proton exchanger 1; ZO-1 = zonula occludens 1; JAM-A = junctional adhesion molecule-A; GAPDH = glyceraldehyde-3-phosphatedehydrogenase; H4K8la = Histone H4 Lysine 8 Lactylation

As shown in Fig. 7A–D, Gln-D significantly down-regulated the mRNA expression of *NHE1*, *Na*^+^*/K*^+^*-ATPase*, *Ca*^*2*+^*/Mg*^*2*+^*-ATPase*, *ZO-1*, *Occludin*, *claudin-1*, and *JAM-A* compared to the Con group, while Gln-D + Gln or SAHA (HDAC inhibitor) could alleviate them (*P* < 0.05). However, both GEN-140 (LDHA inhibitor) and C646 (p300 inhibitor) could block the alleviating effect of Gln (*P* < 0.05). In addition, western blot results (Fig. 7E and F) showed that Gln-D significantly reduced the protein expression of ZO-1, Occludin, and claudin-1 compared to the Con group, while Gln-D + Gln or SAHA (HDAC inhibitor) could alleviate them (*P* < 0.05). Nevertheless, compared with the Gln-D + Gln group, both Gln-D + Gln + GEN-140 (LDHA inhibitor) and Gln-D + Gln + C646 (p300 inhibitor) significantly decreased the protein expression of ZO-1, Occludin, and claudin-1 (*P* < 0.05).

## Discussion

As previously mentioned, malnutrition can reduce the Gln level in mammals [11, 14]. We also discovered that feed deficiency significantly reduced the Gln concentration in serum and rumen epithelium of yaks [27]. The study indicated that Gln deficiency could damage the tight junction integrity in YRECs [15]. Our previous study found that malnutrition significantly enhanced the levels of diamine oxidase, D-lactate, lipopolysaccharide, and histamine in the serum and reduced the expression level of tight junction, indicating an impaired barrier function, thereby decreased the body weight and average daily gain (ADG) of yaks, while Gln supplementation can alleviate them [27]. In addition, Gln supplementation could alleviate malnutrition-induced loss of pre-slaughter weight [31]. This indicates that an exogenous supplement of Gln helps to alleviate the functional damage of ruminal epithelial cells induced by endogenous Gln deficiency. However, further exploration of the underlying mechanism is necessary.

Firstly, nutrient digestibility and rumen fermentation can directly affect the growth of ruminants [32]. In our study, FR had no significant effect on nutrient apparent digestibility, but it significantly reduced the VFA concentrations, such as acetate, propionate, butyrate, and iso-butyrate. This may be a cause of the reduction in body weight and rumen epithelial injury. Because the rumen VFA is the principal source of energy in ruminants, and the low concentration of propionate and butyrate may be detrimental to rumen epithelium development [33, 34]. However, FR + Gln significantly increased the apparent digestibility of DM, NDF, ADF, and CF and the VFA concentrations. An in vitro study found that Gln could produce VFA by anaerobic microbial metabolism, including acetate and butyrate [35]. A previous study had found that dietary Gln supplementation could enhanced the cellulase activity and the relative abundance of unclassified *Peptostreptococcaceae* in the rumen of growth-retarded yaks, thereby enhancing the apparent digestibility of NDF and the VFA concentrations [7]. Our results matched the previous study. Thus, dietary Gln supplementation could enhance the nutrient digestibility and alleviate the abnormal fermentation in the rumen caused by malnutrition, thereby contributing to an increase in growth performance of yaks and the recovery of rumen epithelial damage.

The rumen epithelium plays a vital role as an immune barrier in ruminants and is the main area for nutrient absorption and metabolism [36]. Sodium proton exchanger (NHE), a ubiquitous membrane transport protein, participates in regulating intracellular pH and cell volume [37]. Besides, NHE combines with the actin cytoskeleton, has an indirect connection with tight junctions, and participates in the regulation of gastrointestinal epithelial barrier function [38, 39]. ATPase can facilitate the conversion of ATP into ADP and phosphate ions, releasing energy, and is crucial to maintaining the functional epithelial barrier [40–42]. Thus, NHE and ATPase can indirectly reflect the integrity of the epithelial barrier function. Our results revealed that Gln supplementation alleviated FR-induced and Gln-D-induced reduction in mRNA expression of *NHE1*, *Na*^+^*/K*^+^*-ATPase*, and *Ca*^*2*+^*/Mg*^*2*+^*-ATPase* and enzyme activities of Na^+^/K^+^-ATPase and Ca^2+^/Mg^2+^-ATPase. These results are consistent with the results of tight junctions [27]. And in vitro, Gln alleviated Gln-D-induced TEER reduction in YRECs. This indicates that Gln helps to maintain the integrity of the rumen epithelial barrier.

It has been proven that histone lysine lactylation is widely involved in the metabolic processes in various tissues of yaks [43]. As we all know, histone lysine lactylation is specifically regulated by lactate [18]. In our study, FR or Gln-D significantly reduced the LDHA activity and lactate concentration, thereby decreasing the enzyme activities of p300, HDAC1, and HDAC3, and the level of histone lysine lactylation. The findings of LDHA activity and lactate concentration are consistent with earlier research [23]. Moreover, the low expression of ion channel transport and tight junctions induced by Gln-D in YRECs was partially blocked by the HDAC inhibitor. These results suggest that histone lysine lactylation is involved in Gln-D damage to the YREC barrier function. Subsequently, enhanced glutamine metabolism by Gln supplementation significantly increased lactate concentration and primarily alleviated the reduction in the protein expression level of H4K8la. Nevertheless, one study found that a high-concentration diet elevated histone lysine lactylation by p300 through the upregulation of lactate, inducing an inflammatory response in the mammary gland of dairy cows [44]. This is not in accordance with our findings. The discrepancy could be due to the difference in lactate concentration. Our research showed that Gln-D reduced lactate concentration, while Gln supplementation aimed to restore lactate concentration to normal levels in YRECs. For example, a previous study in pregnant sheep stated that lactate-induced histone lysine lactylation could be dose-dependent in regulating downstream physiological functions, and high-concentration lactate exceeding physiological level was toxic to the endometrium and the embryo [22]. In addition, the p300 inhibitor blocked the effect of Gln in alleviating damage to ion channel transport and tight junctions in YRECs. Thus, the histone lysine lactylation may be involved in Gln alleviating Gln-D-induced damage to the barrier function of YRECs.

## Conclusion

In summary, our results showed that feed restriction inhibited rumen microbial fermentation and reduced the expression of ion channel transport-related genes and the level of histone lysine lactylation, thereby compromising rumen epithelial barrier function. However, on the one hand, Gln supplementation could enhance the nutrient apparent digestibility and rumen microbial fermentation in restricted-fed yak. On the other hand, Gln supplementation could alleviate the reduction of the expression of ion channel transport and tight junctions-related proteins induced by malnutrition by regulating the histone lysine lactylation. Nonetheless, we still lack a control group receiving glutamine alone. Thus, this study only provided a theoretical basis for using Gln to mitigate rumen epithelial damage in yaks induced by malnutrition during the cold season.

## Supplementary Information

Additional file 1: Table S1. Feed compositions and nutrient levels of the basal diet for yaks. Table S2. Primers used in real-time quantitative PCR. Table S3. The information of antibodies (Western blot). Table S4. Search parameters (MaxQuant1.6.14). Table S5. Protein identification overview. Table S6. Histone Lysine lactylation sites. Table S7. Effects of Gln-D and Gln supplementation on histone lysine lactylation sites in YRECs. 

Additional file 2. The original images of western blot analysis.

## Data Availability

The datasets are included in this article and available from the corresponding author on reasonable request.

## Abbreviations

ADF: Acid detergent fiber
ADG: Average daily gain
BCA: Bicinchoninic acid
Con: Control
CP: Crude protein
DM: Dry matter
DMI: Dry matter intake
FBS: Fetal bovine serum
FR: Feed restriction
FR + Gln: Feed restriction + glutamine
GAPDH: Glyceraldehyde-3-phosphatedehydrogenase
GCL: γ‐Glutamyl-cysteine ligase
GDH: Glutamate dehydrogenase
Gln: Glutamine
Gln-D: Glutamine deficiency
Gln-D + Gln: Glutamine deficiency + glutamine
GlnS: Glutaminase enzyme
Glu: Glutamate
GSH: Reduced glutathione
H4K8la: Histone H4 lysine 8 lactylation
HDAC: Histone deacetylase
JAM-A: Junctional adhesion molecule-A
LA: Lactate
LDH: Lactate dehydrogenase
NDF: Neutral detergent fiber
NHE: Sodium proton exchanger
p300: Histone acetyltransferase
PBS: Phosphate buffered saline
PVDF: Polyvinylidene difluoride
SDS-PAGE: Sodium dodecyl sulfate-polyacrylamide gel electrophoresis
SR: Serum replacement
TEER: Transepithelial electrical resistance
TMR: Total mixed ration
VFA: Volatile fatty acid
YRECs: Yak rumen epithelial cells
ZO-1: Zonula occludens 1
α-KG: α-Ketoglutarate dehydrogenase complex
