# Supplementary material for: Glutamine relieves feed restriction-induced ruminal epithelial function damage through histone lysine lactylation in yaks
Source: J Anim Sci Biotechnol. 2025 Dec 18;16:174. doi: 10.1186/s40104-025-01305-7 (PMC12713263; doi:10.1186/s40104-025-01305-7)
Supplement: Supplementary file 1 — Additional file 1: Table S1. Feed compositions and nutrient levels of the basal diet for yaks. Table S2. Primers used in real-time quantitative PCR. Table S3. The information of antibodies (Western blot). Table S4. Search parameters (MaxQuant1.6.14). Table S5. Protein identification overview. Table S6. Histone Lysine lactylation sites. Table S7. Effects of Gln-D and Gln supplementation on histone lysine lactylation sites in YRECs. [file 40104_2025_1305_MOESM1_ESM.docx]

**Table S1** Feed compositions and nutrient levels of the basal diet for yaks (DM basis)

| **Ingredients** | **Content, %** | **Nutrient levels** | **Content** |
| --- | --- | --- | --- |
| Rice straw | 8.00 | NEm, MJ/kg^2^ | 6.38 |
| Oat hay | 27.00 | TDN, % | 66.32 |
| Distiller’s grains | 20.00 | CP, % | 13.13 |
| Corn | 27.45 | NDF, % | 40.02 |
| Wheat bran | 4.72 | ADF, % | 26.48 |
| Soybean meal | 3.60 | Calcium, % | 0.74 |
| Rapeseed meal | 5.85 | Phosphorus, % | 0.46 |
| Soybean oil | 0.27 |  |  |
| Baking soda | 0.36 |  |  |
| NaCl | 0.18 |  |  |
| CaCO_3_ | 0.09 |  |  |
| CaHPO_4_ | 0.18 |  |  |
| Angel active yeast | 0.05 |  |  |
| Premix^1^ | 2.25 |  |  |

*NEm* Net energy for maintenance, *TDN* Total digestible nutrients, *CP* Crude protein, *NDF* Neutral detergent fiber, *ADF* Acid detergent fiber

^1^ The premix provided following per kilogram of the basal ration: Cu 10 mg, Fe 50 mg, Zn 30 mg, Mn 40 mg, Se 0.1 mg, I 0.5 mg, Co 0.1 mg, VA 3000 IU, VD 500 IU, VE 50 IU

^2^ NEm and TDN were the calculated value; the other nutrient levels of the diet were measured values

**Table S2** Primers used in real-time quantitative PCR

| **Gene** | **Primer sequence of forward (5´→3´)** | **Length, bp** | **GenBank ID** |
| --- | --- | --- | --- |
| *NHE1* | F: CGGCTCTCACCACTGGAACTG  R: ACGATGCGGAACTTGTTGATGAAC | 114 | XM_005895895.2 |
| *H^+^-ATPase* | F: GGAAGGCTCGGCGTCTGAAG  R: GCTTGCTCTGGAATTCTTGCTCTC | 93 | XM_005904173.1 |
| *Na^+^/K^+^-ATPase* | F: TGCCTTACGGTTATCGCTACAGAC  R: CTCCTCTTCTTCCTCCTCCTCCTC | 140 | XM_005891531.1 |
| *Ca^2+^/Mg^2+^-ATPase* | F: AGAGATTGAGCAAGAGCGGATAGAC  R: AGCTACCACCTGGCGTTGTTC | 136 | XM_005906321.2 |
| *ZO-1* | F: CCGAATGAAACCGCACACAAACC  R: GTCTCCACGCCACTGTCAAACTC | 107 | XM_014476599.1 |
| *Occludin* | F: GCCTGTGTTGCCTCCACTCTTG  R: CCATAGCCATAACCGTAGCCATAGC | 143 | XM_005889348.2 |
| *claudin-1* | F: CCCGTGCCTTGATGGTGATTGG  R: CATCTTCTGTGCCTCGTCGTCTTC | 110 | XM_005897671.2 |
| *JAM-A* | F: GTGCCTCCATCCAAGCCTACAATC  R: GGCATCTCTACTCCATCCTTGAACC | 134 | XM_010802736.3 |
| *GAPDH* | F: CGGCACAGTCAAGGCAGAGAAC  R: CCACATACTCAGCACCAGCATCAC | 116 | XM_014482068.1 |

*NHE1* Sodium proton exchanger 1, *ZO-1* Zonula occludens 1, *JAM-A* Junctional adhesion molecule-A, *GAPDH* Glyceraldehyde-3-phosphatedehydrogenase

**Table S3** The information of antibodies (Western blot)

| **Item** | **Host** | **Source** | **Catalog No.** | **Dilution for WB** |
| --- | --- | --- | --- | --- |
| Pan Anti-Lactyllysine | Rabbit | PTM Bio (Hangzhou, Zhejiang China) | PTM-1401 | 1:2000 |
| H4K8la | Rabbit | PTM Bio (Hangzhou, Zhejiang China) | PTM-1405 | 1:1000 |
| β-actin | Rabbit | ABclonal (Wuhan, Hubei, China) | AC026 | 1:5000 |
| ZO-1 | Rabbit | ABclonal (Wuhan, Hubei, China) | A0659 | 1:1000 |
| Occludin | Rabbit | ABclonal (Wuhan, Hubei, China) | A12621 | 1:1000 |
| Claudin-1 | Rabbit | Bioss (Beijing, China) | DF6919 | 1:1000 |
| GAPDH | Rabbit | ABclonal (Wuhan, Hubei, China) | AC001 | 1:5000 |

*H4K8la* Histone H4 Lysine 8 Lactylation, *ZO-1* Zonula occludens 1, *GAPDH* Glyceraldehyde-3-phosphatedehydrogenase

**Table S4** Search parameters (MaxQuant1.6.14)

| **Item** | **Value** |
| --- | --- |
| Type of Quantification | Label-free Quantification |
| Datebase | uniprotkb_Bos_grunniens |
| Enzyme | Trypsin |
| Max Missed Cleavages | 2 |
| Peptide Mass Tolerance | 20 ppm |
| Fragment Mass Tolerance | 0.1 Da |
| Variable Modifications | Oxidation (M)；C3H4O2 (K) |
| Fixed Modifications | Carbamidomethyl (C) |
| iBAQ | TRUE |

**Table S5** Protein identification overview

| **Treatment^1^** | **Peptide** | **Identified total protein** | **Identified histone** | **Histone Lysine lactylation sites** |
| --- | --- | --- | --- | --- |
| Con | 1463 | 450 | 18 | 6 |
| Gln | 1510 | 459 | 19 | 5 |
| Gln-D | 1250 | 386 | 16 | 5 |
| Gln-D+Gln | 1495 | 451 | 18 | 5 |

^1^ Con = control group; Gln = glutamine group; Gln = glutamine deficiency group; Gln-D + Gln = glutamine deficiency + glutamine group

**Table S6** Histone Lysine lactylation sites

| **Treatment^1^** | **Histone** | **Annotated Sequence** | **Histone Lysine [K]**  **lactylation sites** |
| --- | --- | --- | --- |
| Con | Histone H4 | SGRGKGGK(1)G | A0A8B9XRN3 lactylation [K8] |
|  | Histone H4 | K(1)AVTAMDVVYALK | A0A8B9YMM1 lactylation [K79] |
|  | Histone H2A | AQGGVLPNIQAVLLPK(1)KTE | A0A8B9XRM7 lactylation [K118] |
|  | Histone H2A | VTIAQGGVLPNIQGVLLPK(1) | A0A8C0ADR5 lactylation [K83] |
|  | Histone H2B | QVHPDTGISSK(1) | A0A8C0AE91 lactylation [K57] |
|  | Histone H1.2 linker | ASGPPVSELITK(1) | A0A8C0ADT0 lactylation [K45] |
| Gln | Histone H4 | SGRGKGGK(1)G | A0A8B9XRN3 lactylation [K8] |
|  | Histone H4 | K(1)AVTAMDVVYALK | A0A8B9YMM1 lactylation [K79] |
|  | Histone H2A | AQGGVLPNIQAVLLPK(1)KTE | A0A8B9XRM7 lactylation [K118] |
|  | Histone H2B | QVHPDTGISSK(1) | A0A8C0AE91 lactylation [K57] |
|  | Histone H3 | FQSSAVMALQEASEAYL  VGLFEDTNLCAIHAK(1) | A0A8B9XSD2 lactylation [K115] |
| Gln-D | Histone H4 | SGRGKGGK(1)G | A0A8B9XRN3 lactylation [K8] |
|  | Histone H4 | K(1)AVTAMDVVYALK | A0A8B9YMM1 lactylation [K79] |
|  | Histone H2A | AQGGVLPNIQAVLLPK(1)KTE | A0A8B9XRM7 lactylation [K118] |
|  | Histone H2A | VTIAQGGVLPNIQGVLLPK(1) | A0A8C0ADR5 lactylation [K83] |
|  | Histone H2B | QVHPDTGISSK(1) | A0A8C0AE91 lactylation [K57] |
| Gln-D+Gln | Histone H4 | SGRGKGGK(1)G | A0A8B9XRN3 lactylation [K8] |
|  | Histone H4 | K(1)AVTAMDVVYALK | A0A8B9YMM1 lactylation [K79] |
|  | Histone H2A | AQGGVLPNIQAVLLPK(1)KTE | A0A8B9XRM7 lactylation [K118] |
|  | Histone H2A | VTIAQGGVLPNIQGVLLPK(1) | A0A8C0ADR5 lactylation [K83] |
|  | Histone H2B | QVHPDTGISSK(1) | A0A8C0AE91 lactylation [K57] |

^1^ Con = control group; Gln = glutamine group; Gln = glutamine deficiency group; Gln-D + Gln = glutamine deficiency + glutamine group

**Table S7** Effects of Gln-D and Gln supplementation on histone lysine lactylation sites in YRECs

| **Histone^1^** | **Histone Lysine [K]**  **lactylation sites** | **FC** | **Up/Down** |
| --- | --- | --- | --- |
| Con vs. Gln |  |  |  |
| A0A8B9XRN3 Histone H4 | lactylation [K8] | 0.82 | down |
| A0A8B9YMM1 Histone H4 | lactylation [K79] | 1.02 | up |
| A0A8B9XRM7 Histone H2A | lactylation [K118] | 1.05 | up |
| A0A8C0AE91 Histone H2B | lactylation [K57] | 0.46 | down |
| Con vs. Gln-D |  |  |  |
| A0A8B9XRN3 Histone H4 | lactylation [K8] | 0.41 | down |
| A0A8B9YMM1 Histone H4 | lactylation [K79] | 0.70 | down |
| A0A8B9XRM7 Histone H2A | lactylation [K118] | 0.49 | down |
| A0A8C0AE91 Histone H2B | lactylation [K57] | 1.23 | up |
| Gln-D vs. Gln-D+Gln |  |  |  |
| A0A8B9XRN3 Histone H4 | lactylation [K8] | 2.24 | up |
| A0A8B9YMM1 Histone H4 | lactylation [K79] | 1.23 | up |
| A0A8B9XRM7 Histone H2A | lactylation [K118] | 1.07 | up |
| A0A8C0AE91 Histone H2B | lactylation [K57] | 0.97 | down |
| Gln-D+Gln vs. Con |  |  |  |
| A0A8B9XRN3 Histone H4 | lactylation [K8] | 0.91 | down |
| A0A8B9YMM1 Histone H4 | lactylation [K79] | 0.86 | down |
| A0A8B9XRM7 Histone H2A | lactylation [K118] | 0.52 | down |
| A0A8C0AE91 Histone H2B | lactylation [K57] | 1.19 | up |

^1^ Con = control group; Gln = glutamine group; Gln = glutamine deficiency group; Gln-D + Gln = glutamine deficiency + glutamine group
