# Supplementary material for: Glutamine relieves feed restriction-induced ruminal epithelial function damage through histone lysine lactylation in yaks
Source: J Anim Sci Biotechnol. 2025 Dec 18;16:174. doi: 10.1186/s40104-025-01305-7 (PMC12713263; doi:10.1186/s40104-025-01305-7)
Supplement: Supplementary file 2 — Additional file 2. The original images of western blot analysis. [file 40104_2025_1305_MOESM2_ESM.docx]

Fig.1 G


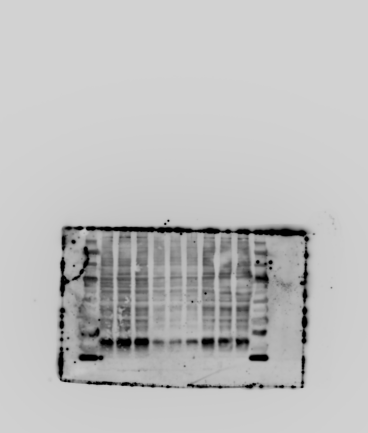
Pan lactylation

Fig.4 E


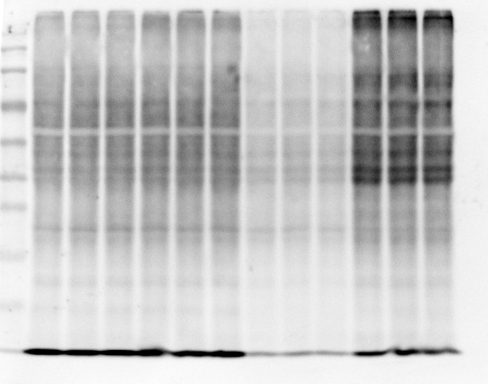
Pan lactylation

Fig.5 E


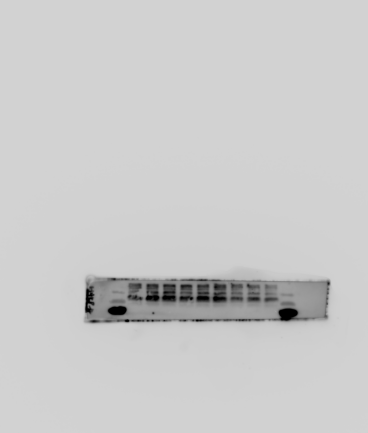
H4K8la
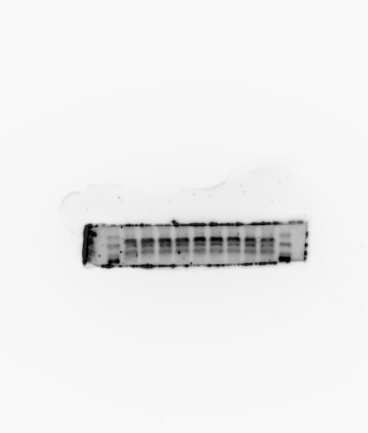
β-actin

Fig.5 J


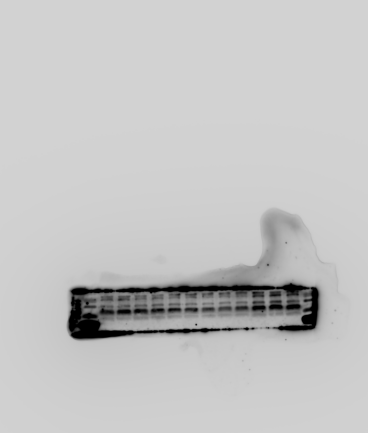
H4K8la
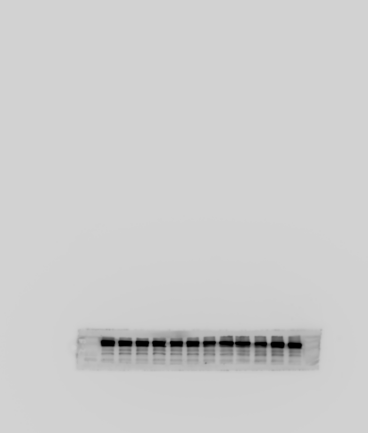
β-actin

Fig.6 E


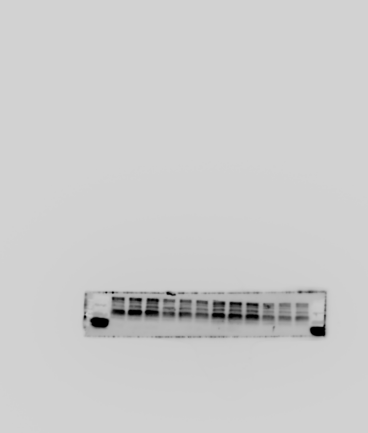
H4K8la
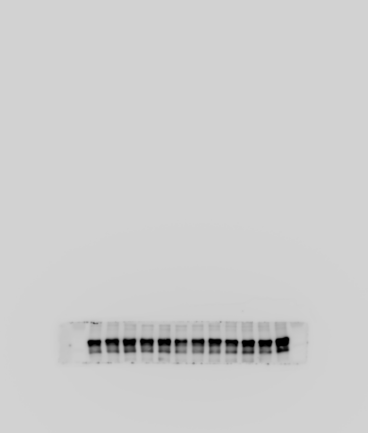
β-actin

Fig.6 F


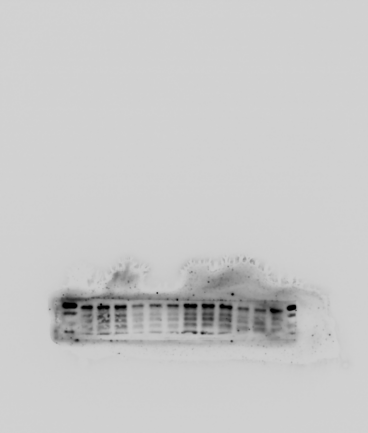
H4K8la
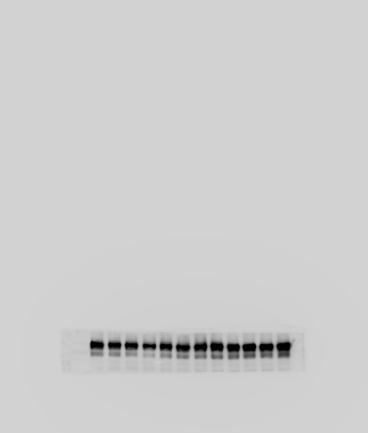
β-actin

Fig.6 G


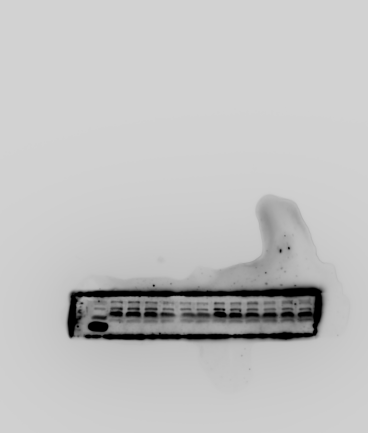
H4K8la
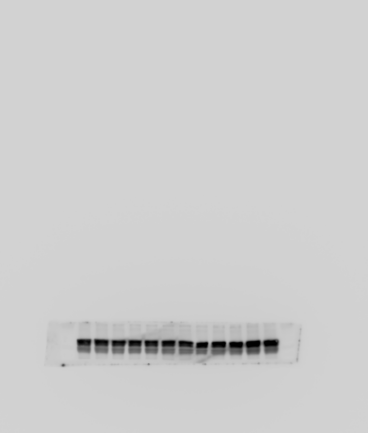
β-actin

Fig.7 E


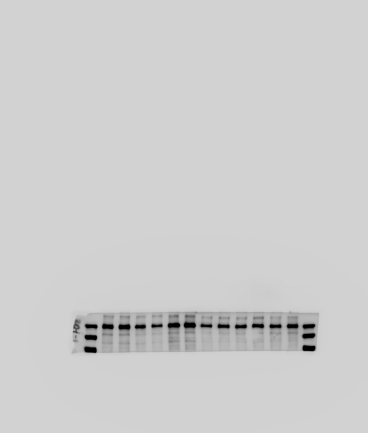
ZO-1（1）
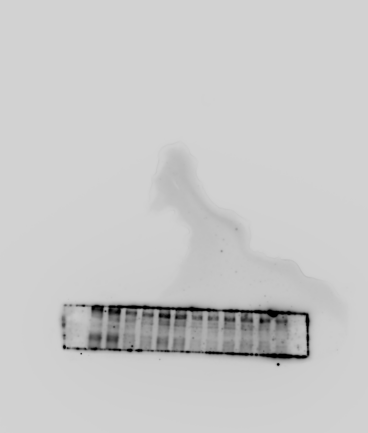
Occludin


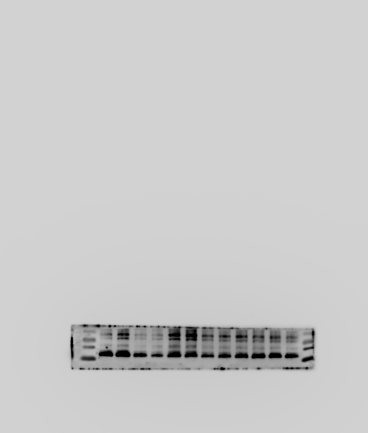
claudin-1（kDa=23）
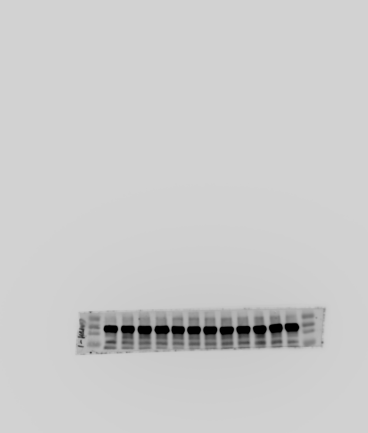
GAPDH
